# P-944. Usefulness of PCR and Culture of Specimens Obtained at Surgery in Patients with Infective Endocarditis

**DOI:** 10.1093/ofid/ofae631.1135

**Published:** 2025-01-29

**Authors:** Nabin K Shrestha, Meghna Nagam, Hannah Wang, Steven M Gordon

**Affiliations:** Cleveland Clinic, Cleveland, Ohio; Cleveland Clinic, Cleveland, Ohio; Cleveland Clinic, Cleveland, Ohio; Cleveland Clinic Foundation, Cleveland, OH

## Abstract

**Background:**

By 2016 it was standard protocol at Cleveland Clinic that patients undergoing surgery for confirmed or suspected infective endocarditis (IE) had cardiac specimens tested by both culture and 16s rRNA PCR and amplicon sequencing (PCR) to verify or accurately identify the causative pathogen. The purpose of this study was to compare the sensitivities and false positive rates, of PCR and culture of cardiac specimens obtained at surgery, among patients with infective endocarditis.

**Methods:**

This was a retrospective cohort study. Episodes of pathologically-proven bacterial endocarditis with a single causative pathogen, among patients who underwent surgery for IE from January 1, 2016, to January 1, 2021 were identified from the Cleveland Clinic Infective Endocarditis Registry. The causative pathogen in the registry is determined by criteria that consider blood culture, histopathological findings, and culture and PCR of surgical specimens. Sensitivities of culture and PCR were compared, as were the false positive rates for PCR and culture among tests that were positive.

**Results:**

Microbiological testing was done on 1326 fresh tissue specimens from 731 episodes of IE among 706 unique patients during the 5-year study period. PCR was significantly more sensitive than culture in detecting the pathogen (79.6% vs. 31.6%, p-value < 0.001). After adjusting for the number of specimens tested per IE episode, the odds of detecting the pathogen were significantly higher for PCR than for culture (OR 10.60, 95% C.I. 8.23 – 13.74, p-value < 0.001). Specimens that tested positive were false positives 1.6% and 10.1% of the time, for PCR and culture, respectively. Among specimens that tested positive, the odds of a positive test result being a false positive were significantly lower for PCR than for culture (13/795 vs 35/313, OR 0.15, 95% C.I. 0.07 – 0.27, p-value < 0.001).

**Conclusion:**

The causative pathogen for IE is much more likely to be identified by PCR of surgical specimens than by culture. Conversely, a positive culture from a surgical specimen is much more likely to be a contaminant than a positive PCR.

**Disclosures:**

**Hannah Wang, MD**, Hologic: Advisor/Consultant

